# Predictive Markers of Honey Bee Colony Collapse

**DOI:** 10.1371/journal.pone.0032151

**Published:** 2012-02-23

**Authors:** Benjamin Dainat, Jay D. Evans, Yan Ping Chen, Laurent Gauthier, Peter Neumann

**Affiliations:** 1 Swiss Bee Research Centre, Agroscope Liebefeld-Posieux Research Station ALP, Bern, Switzerland; 2 Bee Research Laboratory, United States Department of Agriculture-Agricultural Research Service, Beltsville, Maryland, United States of America; 3 Department of Zoology and Entomology, Rhodes University, Grahamstown, South Africa; University of Texas Medical Branch, United States of America

## Abstract

Across the Northern hemisphere, managed honey bee colonies, *Apis mellifera*, are currently affected by abrupt depopulation during winter and many factors are suspected to be involved, either alone or in combination. Parasites and pathogens are considered as principal actors, in particular the ectoparasitic mite *Varroa destructor*, associated viruses and the microsporidian *Nosema ceranae*. Here we used long term monitoring of colonies and screening for eleven disease agents and genes involved in bee immunity and physiology to identify predictive markers of honeybee colony losses during winter. The data show that DWV, *Nosema ceranae*, *Varroa destructor* and *Vitellogenin* can be predictive markers for winter colony losses, but their predictive power strongly depends on the season. In particular, the data support that *V. destructor* is a key player for losses, arguably in line with its specific impact on the health of individual bees and colonies.

## Introduction

Agricultural pollination should integrate wild species, which provide pollination as an ecosystem service, and managed pollinator introduction as crop management practices [1]. Amongst the managed pollinators, the Western honey bee, *Apis mellifera*, is clearly a cornerstone, because pollination of many crops in most parts of the world relies on this species [1]. However, managed honey bee colonies are currently affected by a syndrome corresponding to an abrupt depopulation during winter [2]. Many biotic and abiotic factors are suspected to be involved in this condition, either alone or in combination [2]–[3]. Among them, parasites contribute to weakening colony health, leaving room to secondary infections. In particular, the ectoparasitic mite *Varroa destructor* [4] is now considered to be the main candidate involved in winter colony losses in Europe [5]–[8]. This parasite originates from South-East Asia and has now become widespread across most of the continents [4]. It has been shown that *V. destructor* or its associated microbes can affect the immune system of parasitized bees [9]–[12]. In addition, viral infections linked with *V. destructor* are generally considered as a major cause of bee losses. Indeed, *V. destructor* plays a central role as a mechanical and biological vector of several viruses [12]–[17]. In addition *V. destructor* appears to accelerate the replication of latent viral infections [12], [17]–[20]. Although more than 19 different viruses have been detected in *A. mellifera*, only three have been associated with winter losses on a large scale [5], [21]–[23], namely Deformed wing virus [24], Acute bee paralysis virus [25], [26] and Israeli acute bee paralysis virus [27]. These viruses are positive stranded RNA viruses belonging to the *Iflaviridae* and *Dicistroviridae* families and are suspected to enhance the deleterious action of *V. destructor* on bee colonies by their strong association with this mite [20], [28], [29]. In the United States, IAPV was first identified as a predictive factor for producing CCD symptoms [21]. However, subsequent surveys indicate that this virus was not the main factor responsible for losses but only one of multiple possible factors involved [30], [31]. In Europe, DWV and ABPV are generally suspected to be involved in winter colony losses [5], [22], [23]. Both are transmitted by the mite after feeding on bee pupae or adults [20], [29]. ABPV is highly virulent for bees when injected directly into the hemolymph [25], [29], [32]. On the contrary, DWV is much less virulent and generates typical symptoms of deformed wings only in bees from colonies highly infested with *Varroa* mites [33]. Despite its low virulence for bees, DWV infects a large range of bee tissues and can produce high titers in infected bees, suggesting a potential impact on bee physiology [34], [35].

Another potential candidate involved in colony losses is the microsporidian *Nosema ceranae* [36], [37] although the impact of this parasite on colony health in Europe still remains controversial [5], [38], [39].

Despite the fact that several studies have pointed out the potential involvement of pathogens on colony losses, no common pattern has yet emerged. This is probably due in part to the different parameters present in these studies such as climate, bee races, beekeeping practices or sampling methods. Indeed sampling often consists of bees collected once from either healthy, weak or dead colonies. In this context long term monitoring appears crucial, especially because pathogens causing colony death may have disappeared leaving room for opportunistic infections.

In this study, we aimed to identify predictive markers of winter honey bee colony losses. Although the up- or down-regulation of a marker in a sample may not be related to the principal cause(s) of the disease, such markers would help beekeepers or bee inspectors to set up a reliable diagnostic tool and to standardize bee colony monitoring all around the world. For this purpose we performed a survey of bee colonies in Switzerland over six months and checked for the presence and loads of eleven honey bee pathogens in the samples, as well as the levels of expression of three *A. mellifera* genes involved in bee immunity.

## Materials and Methods

### 1. Experimental design

In summer 2007, 29 queenright colonies were selected from our local bee stocks (predominantly *A. m. carnica*) of similar strength (∼14'000 workers each) at the Swiss Bee Research Centre in Bern, Switzerland. In order to get sufficiently different parasitism levels with the ectoparasitic mite *V. destructor*, 18 colonies were adequately and timely treated in winter 2006–2007 and in summer and fall 2007 against this mite using organic acids following the Liebefelder alternative treatment (twice with formic acid by evaporation using the FAM diffuser in summer and late summer and oxalic acid by droplets in Fall) [40], while the others were left untreated. Both groups were otherwise managed in exactly the same way prior to and during the experiment. In the experiment, the two groups were physically separated by 250 m and one four-storied building to minimize mite movement between colonies (e.g. via drifting and/or robbing; [4], [41]), but nevertheless had similar foraging conditions. Mite infestation levels were monitored weekly in each colony using the natural mite fall method [4], [42], distinguishing colonies with high and low mite infestations. Pooled worker samples (N = 100) were collected alive in the brood nest in summer (18-08-2007) at the beginning of the experiment and in fall (22/11/2007). These two samplings were called “Summer” and “Fall”. Another sampling, “Winter”, was ultimately performed during winter but at different times according to the destiny of the colony: in colonies which survived winter, bees were collected on 28/01/2008 while for the colonies that did not survive, this sampling was done just before collapsing from 30/11/2007 to 28/01/2008. Colony-level traits (areas covered with bees, number of cells with open, sealed brood, honey and pollen) were estimated in dm^2^ every 3 weeks from September until the end of October and at the beginning of March until May using the Liebefelder standard method [43].

### 2. Molecular approaches

Pools of 100 workers were collected alive from the brood nests of each colony and immediately frozen at −20°C. For total RNA extraction, bees were first homogenized in 20 ml of Tris-NaCl buffer (Tris 10 mM; NaCl 400 mM; pH 7.5). An aliquot of 50 µl of the homogenate was used for RNA extraction with the NucleoSpin RNA II Kit® (Macherey-Nagel) following the recommendations of the supplier. Then, cDNA was immediately processed using M-MLV reverse transcriptase (Invitrogen) and random hexamers [44]. These samples were checked for the presence of eight honey bee viruses (DWV, ABPV, IAPV, Chronic bee paralysis virus, Kashmir bee virus, Black queen cell virus, Sacbrood virus and Slow bee paralysis virus), using a qualitative PCR assay [31], [44], [45] with viral cDNA for positive control and water for the negative control. DWV, BQCV and ABPV positive samples were further processed for the quantification of viral titers using quantitative PCR (qPCR). The microsporidians *N. ceranae* and *N. apis* were quantified as well in each sample using qPCR [31]. In parallel, the expression levels of *A. mellifera* transcripts including *vitellogenin*, *eater* and *hymenoptaecin* were monitored using qPCR [46]. In order to normalize the data according to the amount of RNA in the sample, analysis of the *β-actin* gene was performed in parallel for each sample [47]. For all the targets except DWV, normalization was done using the comparative quantification method (delta CT method) [48]. DWV loads in samples were quantified by absolute quantification method using standard curves made of serial dilutions of known amounts of the amplicons [49] and presented as equivalent copies of DWV genome. All qPCR reactions were conducted using a thermal profile of: 50°C (2 min) then 95°C (10 min) followed by 40 cycles of 95°C (15 s), 60°C (1 min). Because qPCR assays were performed using SYBR-Green (Eurogentec), a melting curve was performed at the end of each run to ascertain the amplification of the target.

### 3. Data analyses

The bee colony samples were divided into two groups according to their winter survival. The first group (DC: dead colonies) consisted of 13 colonies which died during winter; most of these (11 out of 13) belonged to the set of colonies that received no treatment against *V. destructor*. The second group (SC: surviving colonies) included the 16 colonies that survived winter and coincided with the colonies that received a treatment against the mite. The variation estimates of transcript abundance of the studied variable between different groups were evaluated by using two tailed t-tests and non-parametric Kruskal–Wallis test as appropriate. Since DWV values covered a wide range, the data were transformed as the log10 DWV. For survival analysis, a Kaplan–Meier survival analysis was performed and used to compare the groups using Mantel-Hansel tests. Linear Models were also performed using Standard Least squares fitting in the Fit Model platform of JMP software to test the effects of colony status when nested within season. To visualize the results, regression diagnostics were performed with the model leverage plot. This allowed us to test if the variables are predictive on overwintering abilities and also to check the influence of each point on each hypothesis test. Multivariate Spearman correlations were performed between the variables (Pathogens and physiological markers). *P*-values below 0.05 were considered significant. The analyses were performed using Systat 12® and JMP® software.

## Results

### 1. Bee population measurements and timing of colony collapse

**Figure 1:**
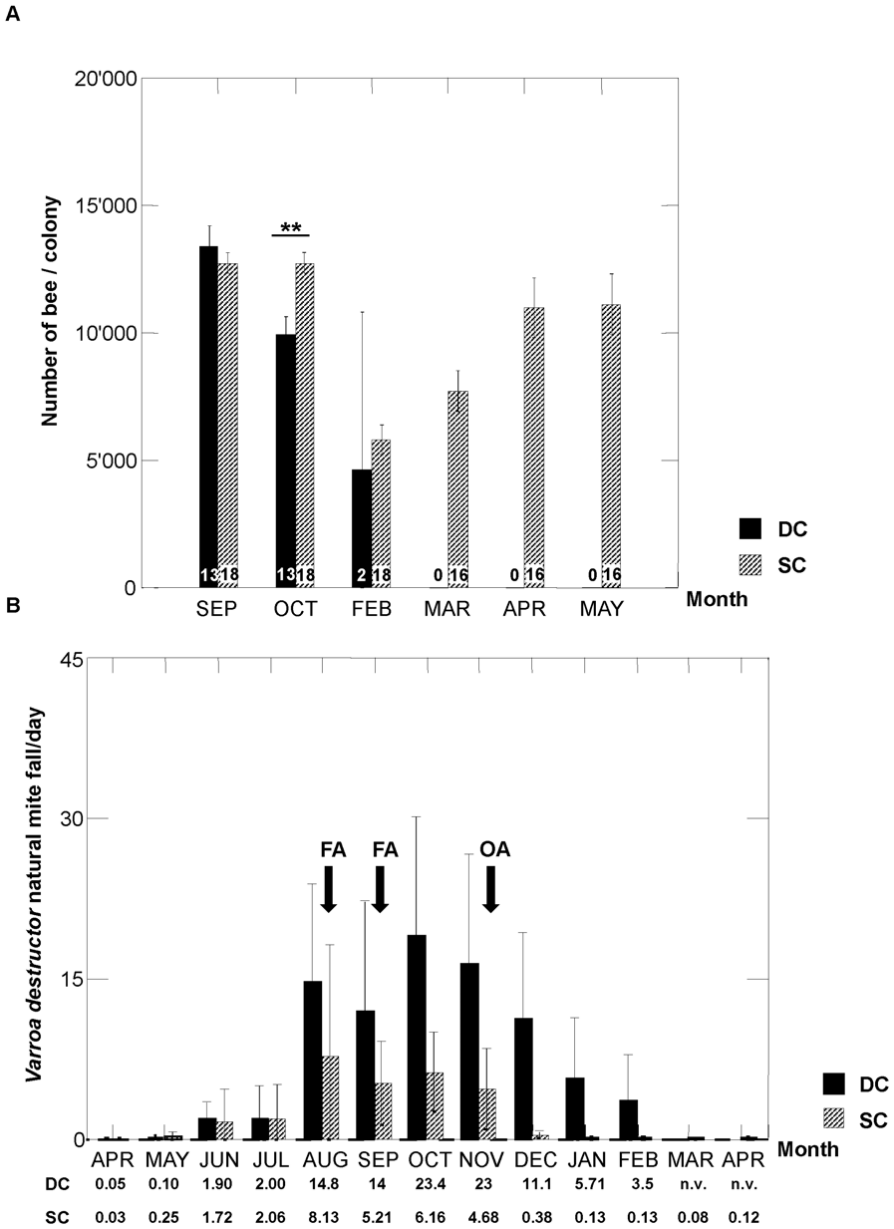
Colony strength and *V. destructor* natural mite fall. A) Colony strength (Y-axis) by sampling month in 2007/2008 (X-axis) for the colonies that died during winter (DC, black boxes) and the ones that survived (SC, grey boxes). P value is indicated (Mann-Whitney test): **P<0.01. Number of live colonies is given (N). B) Natural average *V. destructor* mite fall in 2007/2008 on the hive bottom boards per day per colony (Y-axis) for the two groups Dying Colonies (DC) and Surviving Colonies (SC) over the experimental period. (X-axis) Infestation levels [average ± SD] are shown for each month and timing of treatments (OA = Oxalic Acid, FA = Formic acid) is indicated by arrows (n.v. = no value).

While the colony size did not differ significantly in September between SC and DC groups (Figure 1; Mean: 12743.75 bees and 13376.92 respectively, Mann-Whitney test P = 0.07), a significant decrease in population was observed in October (29/10/2007) in the group of colonies that died during winter (Mann-Whitney test, P<0.01, Figure 1 A). In the untreated group most of the colonies collapsed during December 2007 (N = 9) and two out of 11 died before February 2008. Two colonies properly treated against *V. destructor* mites died during winter but later in the season (end of February) and obviously because of queen failure (these colonies became drone layers). The rest of the colonies did develop with an increased population as expected in this season (Figure 1 A). The *V. destructor* infestation levels and timing of treatments are indicated in Figure 1 B. After each mite treatment, we had considerable treatment Varroa fall confirming the treatment efficacy. From August to September, the natural daily mite fall in the surviving colonies decreased from [average ± SE] 8.13±1.95 to 5.21±0.5 per day with 0.377±0.089 after the last oxalic acid treatment.

### 2. Parasites and Pathogens

**Figure 2:**
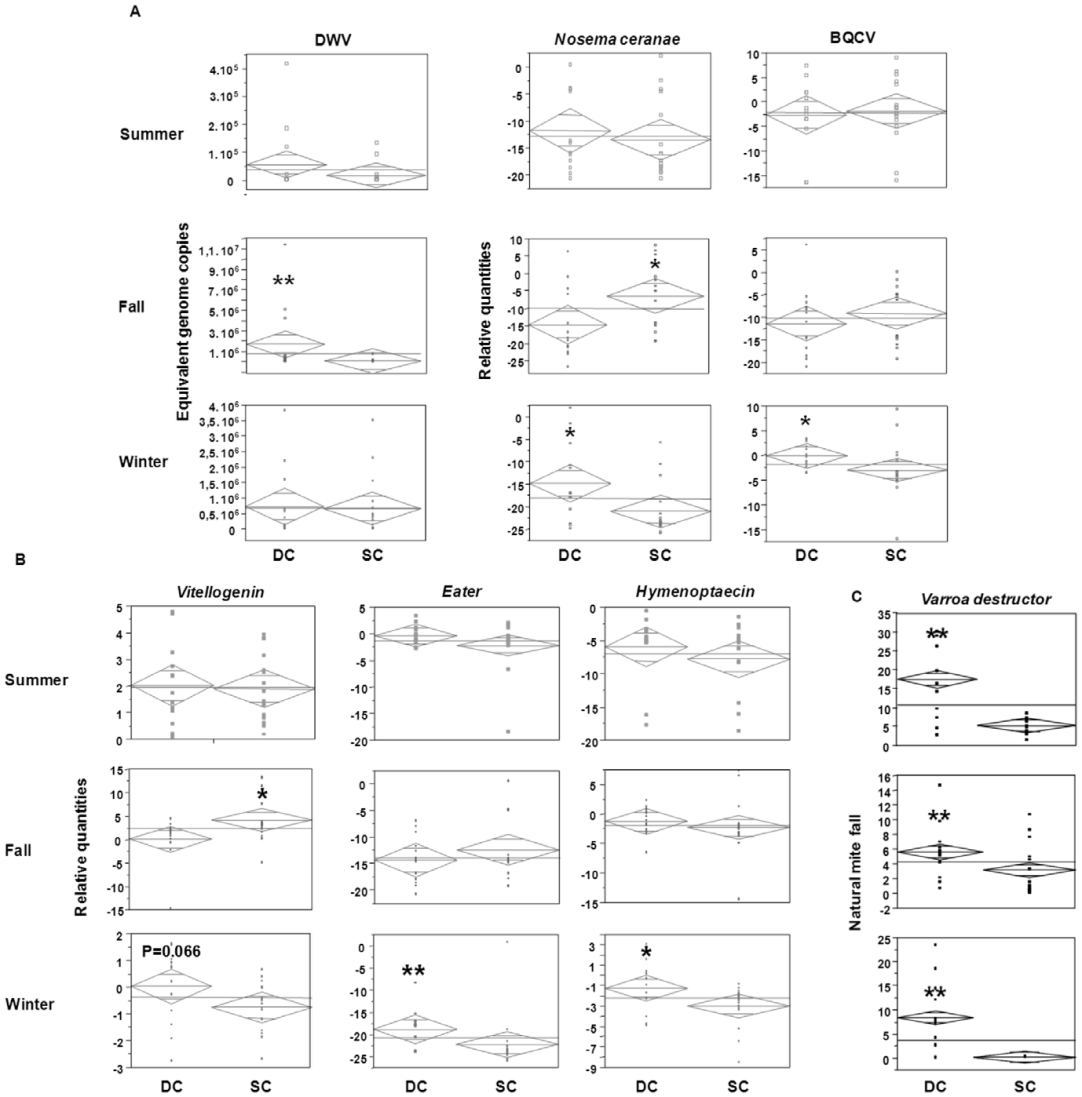
Seasonal variability in Summer, Fall and Winter. A) Pathogens B) Gene expression profiles C) *V. destructor*. The Y-axis shows the relative quantities except for DWV, where it shows equivalent genome copies and the X-axis displays the groups Dying Colonies DC (N = 13) and Surviving Colonies SC (N = 16). Significant differences (Two tailed t-test) are indicated with * = P<0.05 and ** = P<0.01.

The number of *V. destructor* mites collected during the course of the experiment in the DC group exceeded those collected in the SC group (Figure 1 B and 2 C). The mite level in the DC increased constantly until October before decreasing slightly thereafter. Mite loads in the surviving colonies dropped substantially from October to the winter sample.

Only five honey bee viruses (DWV, BQCV, ABPV, SBV and SPV) were detected in the experimental colonies (Table 1). The viruses CBPV, KBV and IAPV were not detected. DWV had 56.25% prevalence for SC and 61.5% for DC in summer. Almost all colonies were positive for this virus in fall (93.75% for SC and 100% for DC). Conversely, BQCV and SBV displayed a lower prevalence during the cold season, with no detectable SBV sample in the fall sample. We failed to detect SPV in summer and this virus was identified in less than 20% of the samples collected in fall in both DC and SC groups. No SPV could be detected in the bees that survived winter although the virus was detected in three colonies of the DC just before colony collapse. Although all colonies were positive for *N. ceranae*, *N. apis* could not be detected. The prevalence of *N. ceranae* was equivalent between the groups in all seasons and ranged between 18.75% and 50% (Table 1). No distinction could be made in terms of numbers of the detected investigated pathogens between the SC and DC groups in any season as shown in table 1 (Mann-Whitney U-tests, Summer: P = 0.729; Fall: P = 0.854, Winter: P = 0.359).

**Table 1:** Proportion of colonies with detectable levels of pathogens as measured by PCR and qPCR in surviving (SC) and dying (DC) colonies.

Season	Status	Colonies	DWV (%)	BQCV (%)	ABPV (%)	SBV (%)	SPV (%)	*N. ceranae* (%)	Nr Path
**Summer**	**DC**	13	61.5	84.6	7.7	15.4	0	38.5	2.08±0.64
	**SC**	16	56.3	87.5	0	43.8	0	31.3	2.19±1.17
**Fall**	**DC**	13	100	53.8	7.7	0	15.4	30.8	2.08±1.04
	**SC**	16	93.8	50	0	0	18.8	50	1.94±0.85
**Winter**	**DC**	13	100	100	0	7.7	23.1	38.5	2.69±0.95
	**SC**	16	100	93.8	0	25	0	18.8	2.38±0.62

Total numbers of pathogens (Nr Path) are summarized in the last column (±SD). No significant differences in pathogen numbers were found between Live and Dead in any season (Mann-Whitney test, Summer P = 0.729; Fall P = 0.854, Winter P = 0.359). CBPV, KBV, IAPV and *N. apis* were not found in any colony.

### 3. Expression levels of bee pathogens and genes related to the bee immunity

As none of the qualitative PCR analysis showed significant differences between surviving colonies and colonies that died during winter (Table 1), we conducted quantitative analyses from samples collected in summer, fall and winter in order to point out potential differences. Because winter samples from the DC group were collected just before collapsing and therefore at different time points during winter, data analyses are presented separately for DC and SC groups (Fig. 2). We measured the expression levels of three pathogens (DWV, BQCV and *N. ceranae*) based on their high prevalence in our samples and their potential pathogenic effects at the colony level. ABPV was excluded from these assays because only three samples were found infected by this virus. In addition, we completed these analyses by measuring in parallel the expression levels of three *A. mellifera* genes involved in bee immune defenses (*hymenoptaecin* and *eater*) or bee life expectancy (*vitellogenin*).

#### Seasonal variation

**Figure 3:**
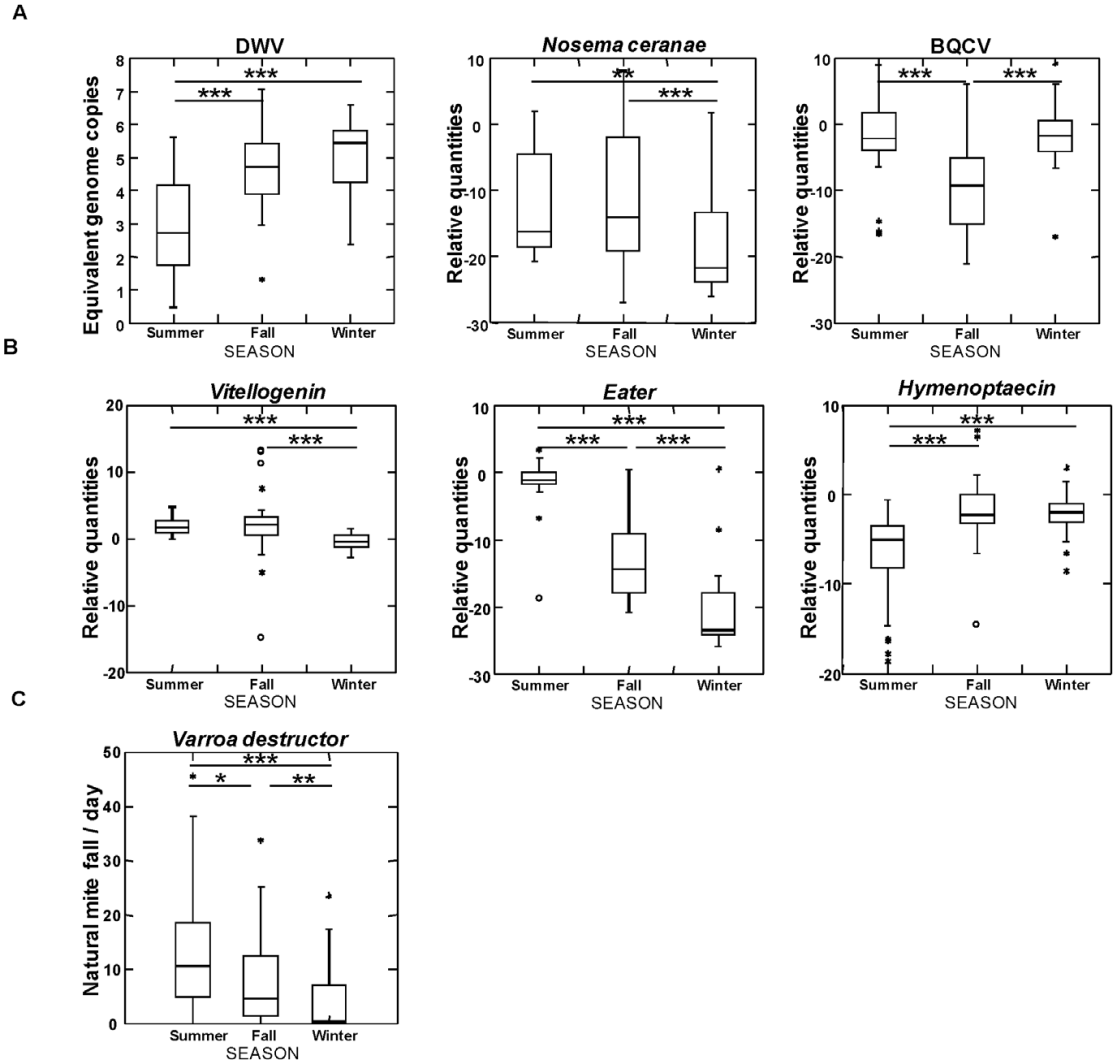
Overall seasonal variability of colonies (N = 29) from Summer to Winter. A) Pathogens B) Genes C) *V. destructor*. The Y-axis show the relative quantities except for DWV, where it shows equivalent genome copies [log10]. The seasons summer, fall and winter are shown on the X-axis. (Mann-Whitney test with Bonferoni correction,* P<0.05; **P<0.01; ***P<0.001).

Without considering the outcome of the colonies (death or survival during winter), seasonal variation was observed between summer and fall (Figure 3). DWV expression was higher in fall than in summer (P<0.001), but conversely BQCV titers were lower in fall than in summer (P<0.001). *N. ceranae* quantification showed equivalent titers between summer and fall (P = 0.479). *Vitellogenin* mRNA titers remained stable between summer and fall (P = 0.544) *while hymenoptaecin* mRNA levels were higher in fall than in summer (P<0.001). Conversely, *eater* expression was significantly reduced in fall compared to summer (P<0.001).

Such comparisons between fall and winter were more difficult to address, especially for colonies that died during winter, because the sampling was done just before the population decline and thus for this group, winter sampling encompassed several weeks, from December, when most of these colonies died, to January. In contrast, all samples were collected by the end of January in colonies that survived winter. The results are presented in table 2. In the group that died during winter, significant differences could be observed between samples collected in fall and those collected upon collapsing but only for BQCV which displayed increasing levels (P<0.001) and the *eater* gene for which expression conversely decreased (P<0.05). The levels of *N. ceranae* remained stable (P = 0.94). In the surviving group, significant variations were observed between fall and winter: *N. ceranae* levels decreased (P<0.001) as well as *eater* (P<0.001) and *vitellogenin* (P<0.001) mRNA levels. In contrast, DWV increased (P<0.05) as well as BQCV (P<0.05). Levels of *hymenoptaecin* were stable (P = 0.981).

**Table 2:** Seasonal variability for all colonies together from Summer to Winter for pathogen loads, *Varroa destructor* (Vd) and expression levels for *vitellogenin* and immune genes.

TARGET	SUMMER TO FALL		FALL TO WINTER	
	DC	SC	DC	SC
***Vd***	**→**	**→**	**→**	**↓↓↓**
***DWV***	**↑↑↑**	**↑**	**→**	**↑**
***BQCV***	**↓↓**	**↓↓**	**↑↑↑**	**↑**
***Nosema ceranae***	**→**	**↑**	**→**	**↓↓↓**
***Vitellogenin***	**→**	**→**	**→**	**↓↓↓**
***Eater***	**↓↓↓**	**↓↓↓**	**↑**	**↓↓↓**
***Hymenoptaecin***	↑↑↑	↑↑	**→**	**→**

Data are grouped by colony status after Winter (SC = Surviving colonies, DC = Dying colonies). (Mann-Whitney test :↑** = **P<0.05; ↑↑ or ↓↓** = **P<0.01; ↑↑↑ or ↓↓↓** = **P<0.001; **→ = **No significant difference).

#### Comparisons between DC and SC groups

Significant variations were observed in the fall between DC and SC groups, but only for DWV, *N. ceranae* and *vitellogenin* mRNA (Figure 2). No differences in any of the targets analyzed here were observed in summer between the two groups. DWV loads were higher in fall in the collapsing colonies than in the surviving ones (P<0.01). In fall, colonies that collapsed during winter displayed lower levels of *N. ceranae* than surviving ones (P<0.05).while in the summer *N. ceranae* levels were similar in both DC and SC groups (P = 0.483).Variations in BQCV titers between SC and DC groups were only observed in winter (P<0.05). While no *vitellogenin* differences could be observed in summer between both SC and DC groups, this gene displayed significantly higher expression levels in fall in the surviving colonies than in the DC group (P<0.05).

### 4 Correlations between markers

**Figure 4:**
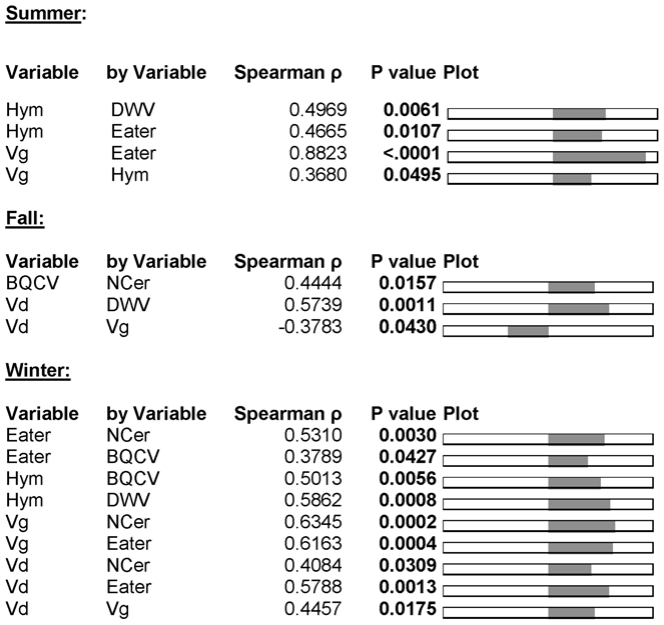
Spearman rank correlations for the different variables who showed significant P values (P<0.05, indicated in bold) for the three seasons summer, fall and winter (N = 29 colonies). The variables shown are: Hym = Hymenoptaecin, Vg = Vitellogenin, Eater, NCer = *Nosema ceranane*, DWV, BQCV and Vd = *Varroa destructor*.

Correlations between the six markers were observed (Figure 4). In summer, DWV *vs. hymenoptaecin* (r_s_ = 0.50, P<0.01), *hymenoptaecin vs. eater* (r_s_ = 0.46; P<0.05), *vitellogenin vs. eater* (r_s_ = 0.88, P<0.001) and *vitellogenin vs. hymenoptaecin* (r_s_ = 0.37, P<0.05) showed significantly positive correlations. In fall, these correlations were not observed anymore but BQCV showed a positive correlation with *N. ceranae* (r_s_ = 0.44, P<0.05). In fall, the number of *V. destructor* mites collected on bottom boards was significantly correlated with both DWV (positively) and *vitellogenin* (negatively) expression levels (r_s_ = 0.57, P<0.05 and r_s_ = −0.37, P<0.05, respectively). In contrast, *V. destructor* correlated positively in winter with *N. ceranae* (r_s_ = 0.41, P<0.05), Eater (r_s_ = 0.58, P<0.01) and *vitellogenin* (r_s_ = 0.45, P<0.05). Full data are presented in Figure 4.

### 5. Identification of predictive markers for colony collapse

Using a linear model (see M&M) the results show that over all seasons, DWV (P<0.05), *V. destructor* (P<0.001), *N. ceranae* (P<0.001) and *vitellogenin* (P<0.001) could be considered as predictive markers for winter losses. This was not the case for BQCV (P = 0.467), *eater* (P = 0.173) and *hymenoptaecin* (P = 0.376) which displayed few variations between DC and SC groups either in summer or in fall. However, the model showed that there is a significant seasonal impact on the expression of these markers (DWV: P<0.05; *V. destructor*: P<0.01, *N. ceranae*: P<0.05 and *vitellogenin*: P<0.01). Then in summer only *V. destructor* (P<0.01) could be considered as a significant predictive marker while in fall, DWV (increased; P<0.01), *V. destructor* (increased; P<0.01), *N. ceranae* (decreased; P<0.05) and Vitellogenin (decreased; P<0.05) could be considered as significant predictors of colony collapse.

## Discussion

Here we showed that both *V. destructor* and DWV are strong predictive markers for honey bee colony death during winter.

Among the high number of microorganisms which are coexisting with honey bee colonies, most are opportunistic and induce troubles under as-yet undefined environmental conditions. It is then crucial for establishing a proper diagnosis of bee diseases to be able to distinguish between a normal situation and a pathogenic one. This can be partly achieved by measuring the expression levels of pathogens in honey bees using quantitative techniques, although data can only be recorded when clinical signs are detected or after colony collapse. Here we present a novel approach, which consists of identifying markers which could predict the destiny of the colony during winter. These markers may help to establish reliable diagnostic tools in relation with field observations, and ultimately to identify the causes of colony mortality.

### 1. *Varroa destructor* is strongly associated with colony collapse during winter

In our assay, all of the colonies which were left untreated died during winter, while only two colonies collapsed despite proper treatment. These two colonies collapsed late in the season (in February) in comparison with the majority of the other colonies which died before the end of December. These two colonies had an over-abundance of male bees, arguably a sign of failing queen fecundity. Colonies that died during winter had significantly more mites than the surviving group either in summer, fall or winter. *V. destructor* loads were thus very predictive of colony death and, in fact, provided the only predictive marker in summer. The mite level in the colonies which died during winter increased to reach a peak in October and decreased thereafter following the drop in colony size.

### 2. DWV is a predictive marker of colony collapse

Several bee viruses were identified. Apart from SPV which was only detected in highly mite-infested colonies here and rarely in previous surveys of European bees [45], [50], DWV, SBV and BQCV are commonly detected in western honey bee colonies all around the world, most of the time in the absence of clinical signs, although these viruses can be pathogenic under favorable environmental circumstances [20]. We found a significant increase of DWV titers between summer and fall. This result is consistent with previous reports showing an increase in DWV titers in fall [49], which probably reflects the close link between DWV and *V. destructor* since mite numbers climb rapidly from summer to fall. It is also illustrated with the significant positive correlation in fall between *V. destructor* and DWV. This mite is indeed an efficient vector of DWV, transmitting it upon feeding on bee pupae or during the phoretic phase of its biological cycle on adult bees [14], [51], [52]. The mite seems also competent for replication of the virus thereby increasing DWV prevalence and titers in mite infested colonies [33], [53]. Proper treatments of bee colonies against *V. destructor* drastically reduced DWV titers in colonies supporting arguments that this virus is not efficiently horizontally transmitted in the absence of mites [54] and that vertical transmission routes are unlikely to generate heavy loads in bee progeny. We found higher DWV titers in collapsing colonies than in the surviving group suggesting that this virus might be involved in the process of collapsing. DWV was shown to replicate in various bee tissues including fat body, a key tissue involved in many physiological processes including immune defenses [34], [55]. In particular, the fat body is the site for production of the egg yolk protein vitellogenin [34], [56]. Vitellogenin is involved in immunity and ageing through hormonal regulatory pathways and is therefore a common molecular marker for the overall health and lifespan of individual bees [57]. One can hypothesize that DWV replication impairs the expression of vitellogenin in fat body cells, which could explain why we found significantly less *vitellogenin* mRNA titers in collapsing colonies than in surviving ones. In addition, *V. destructor* infestation levels and *vitellogenin* were significant negatively correlated in fall. The data agree well with a previous study showing a reduction of Vitellogenin titers in mite infested workers [58], although DWV quantification was not addressed in the prior study. Put together, this reinforces the hypothesis of an impact of DWV on fat body function.

### 3. *Nosema ceranae* titers are higher in healthy colonies

*N. ceranae* is a microsporidian intracellular parasite suspected to have replaced its common relative *N. apis* during the last decade [59], [60], although there are some reports showing that *N. ceranae* has been present in western honey bees for greater than twenty years [59], [61]. This parasite replicates in the bee gut and is therefore a potent pathogen of bee colonies even if its pathogenicity has only been observed in experimental conditions. These infections are very common in the absence of clinical symptoms and recent reports have shown that this parasite is not involved in bee colony collapses in Europe [5], [39] except in Spain [36], [62]. Our results support that *N. ceranae* is not involved in colony collapse, because no significant differences were observed between colonies that died during winter and those which survived. Likewise, we did not observe an increase of *N. ceranae* titers from summer to fall, even in December when colonies were about to die. No *N. apis* were detected in any sample in spite of the fact that this species has a lower temperature preference than *N. ceranae* and might therefore be favored in temperate climates such as in Switzerland [60], [63]. In a case study in Switzerland, *N. apis* was detected only in mixed infections with *N. ceranae* [64], consistent with these results.

### 4. Immune genes are upregulated in dying colonies

We monitored the expression of three *A. mellifera* genes involved in humoral and cellular immune defenses to identify physiological responses that may occur before colony collapse. In workers, in addition to being the precursor of royal jelly proteins produced by nurses to feed the larvae, the egg yolk glycoprotein vitellogenin displays multiple functions affecting important physiological pathways [65]. Among these, it has been shown that vitellogenin plays a role in bee immunity as a zinc transporter [66]. Hymenoptaecin is an antimicrobial peptide, which is highly expressed in the bee hemolymph after challenge with bacterial infections [48], [67]. Eater is a major phagocytic receptor for a broad range of bacterial pathogens in *Drosophila* [68] and its homolog was identified in the honey bee genome [48], [69].

The analysis of such markers from pooled individuals is complex, especially for samples collected in summer, which contained a mix of workers of different ages, because the expression of these genes might also vary according to the age-related task of worker bees. In foragers it has been shown that the vitellogenin titers as well as the number of hemocytes were strongly reduced compared to the nurse bees [70], [71]. Furthermore, no data are available concerning the gene expression patterns of winter bees. Despite that, we observed significant seasonal variations among these genes. While *vitellogenin* expression remained stable from summer to fall, *hymenoptaecin* and *eater* displayed opposite expression patterns in both groups of colonies (surviving and non surviving colonies). The rapid decrease of *eater* from summer to fall suggests that hemocyte numbers are reduced in winter bees while humoral immune responses are activated. Data analyses from fall to winter in samples collected from colonies that did not survive winter (mostly collected in December before collapsing) showed no variation in either *vitellogenin* or *hymenoptaecin* mRNA levels while *eater* displayed a slight increase. In contrast, samples collected from colonies that survived winter showed a very high increase of both *vitellogenin* and *eater* transcripts while *hymenoptaecin* kept stable. Since these samples were collected at the end of January, these results may point out a different physiological state of bees collected in winter clusters two months apart. One can hypothesize that *V. destructor* – DWV – *N. ceranae* may induce different immune signaling transduction pathways than the pathway, leading to the immune transcript hymenoptaecin [48]. As such, none of the immune genes could be identified as significant predictive markers, while vitellogenin does seem to be a viable marker.

### 5. *Varroa destructor* and DWV are strong predictive markers for colony collapse during winter

From the putative investigated markers, four of them were shown to be good predictive ones but seasonal. Among them, *V. destructor* and DWV were already identified during summer as strong predictive markers for collapsing during winter. As winter bees are reared as soon as mid-summer until mid-September in Switzerland [72], these data suggest that *V. destructor* infestations levels in colonies should be estimated by beekeepers as soon as summertime in order to anticipate winter colony losses. Therefore, recording of DWV loads might not be as suitable since DWV was shown to be a strong marker of winter colony collapse in fall but not in summer.

In general, the results presented here are in line with those of several studies showing that *V. destructor* and DWV are associated with colony losses in winter [5], [6], [8], [22], and in contrast to analyses of the enigmatic Colony Collapse Disorder in the U.S., for which mite numbers were a poor correlate with CCD risk [31].

### 6. Conclusion

This study provides evidence that *Varroa destructor* is a key player for winter colony losses and highlights the urgent need for efficient treatments against this parasite. The data suggest an indirect effect of mite infestation on honeybee overwintering abilities through the promotion of opportunistic viral infections, which eventually lead to the impairment of critical physiological functions. The knowledge gathered in this work will help to improve our understanding of bee losses, standardize methods for biomarkers of disease and finally to mitigate causes of bee declines.
